# Lewy Body-Associated Proteins A-Synuclein (a-syn) as a Plasma-Based Biomarker for Parkinson’s Disease

**DOI:** 10.3389/fnagi.2022.869797

**Published:** 2022-05-12

**Authors:** Xuemiao Zhao, Haijun He, Xi Xiong, Qianqian Ye, Feifei Feng, Shuoting Zhou, Weian Chen, Kai Xia, Shuangjie Qian, Yunjun Yang, Chenglong Xie

**Affiliations:** ^1^Department of Radiology, The First Affiliated Hospital of Wenzhou Medical University, Wenzhou, China; ^2^Department of Neurology, The First Affiliated Hospital of Wenzhou Medical University, Wenzhou, China; ^3^Key Laboratory of Alzheimer’s Disease of Zhejiang Province, Wenzhou, China; ^4^Institute of Aging, Wenzhou Medical University, Wenzhou, China; ^5^Oujiang Laboratory, Wenzhou, China

**Keywords:** α-synuclein, Lewy body-associated proteins, biomarkers, diagnosis, Parkinson’s disease

## Abstract

**Introduction:**

To explore the combined diagnostic value of plasma Lewy body-associated proteins (p-Asyn at ser129, total α-syn, and oligomeric α-syn) for the diagnosis of PD versus healthy controls (HCs) and other PD syndromes (PDs), as well as clinical characteristics prediction.

**Methods:**

This study included 145 participants: 79 patients with PD, 24 patients with PDs, and 42 HCs. A panel of plasma levels of p-Asyn, total α-syn, and oligomeric α-syn was measured by enzyme-linked immunosorbent assay (ELISA). The primary outcome was the discriminative accuracy of the combined three plasma biomarkers for PD.

**Results:**

The mean age was 65.43 (SD, 7.467) in the control group, 64.49 (SD, 8.224) in participants with PD, and 69.25 (SD, 7.952) in PDs. The plasma Lewy body-associated protein levels were significantly higher in patients with PD than in age-matched HCs, However, there was no difference in patients with PD and PDs. Of note, a combination of plasma p-Asyn, total α-syn, and oligomeric α-syn was a better biomarker for discriminating PD from HCs, with an AUC of 0.8552 (*p* < 0.0001, 95%CI, 0.7635–0.9409), which was significantly higher than plasma p-Asyn (ΔAUC, 0.1797), total α-syn (ΔAUC, 0.0891) and oligomeric α-syn (ΔAUC, 0.1592) alone. Meanwhile, Lewy body-associated proteins had no connections between different motor stages and dementia performances.

**Conclusion:**

Our results suggested that plasma Lewy body-associated proteins, may serve as a non-invasive biomarker to aid the diagnosis of PD from HCs. In addition, increased plasma Lewy body-associated proteins were not associated with the progression of motor and non-motor symptoms.

## Introduction

Parkinson’s disease (PD) is mainly characterized by pathological aggregation of insoluble α-synuclein (a-syn) in intracellular deposits termed Lewy bodies (LBs) in midbrain nigral neurons/striatum and a long pre-clinical neurodegeneration phase begins years before the appearance of motor manifestations including resting tremor, bradykinesia, rigidity, and postural disturbance (Bloem et al., 2021). A -syn involves physiologically in the management of dopamine synthesis, neural differentiation, synaptic plasticity, etc. (Bendor et al., 2013). Nevertheless, α-syn pathology spreads between tissues and neurons by mechanisms that are still not fully clear. Genetic studies uncovered the point mutations or locus duplications in the a-syn encoding gene (SNCA) were related to autosomal dominantly inherited PD (Stefanis, 2012). Hence, numerous studies aimed to reveal the possible role of a-syn as a candidate biomarker for PD in cerebrospinal fluid (CSF) as well as in blood (Foulds et al., 2011; van Rumund et al., 2019).

Until now, diagnostic tools in PD include structural and functional imaging and the levels of CSF α-syn, which is in direct contact with the brain. Undoubtedly, obtaining CSF routinely in clinics is not an ideal way due to the injury of lumbar punctures and difficulties in repeated collections (Seino et al., 2019). Hence, less invasive biomarkers such as blood are preferable to CSF markers and several studies have assessed the diagnostic value of α-syn in blood, but the data have been conflicting arising from the diversity of research methods and a-syn production source variety (Foulds et al., 2013; Chang et al., 2019; Niu et al., 2020). Meanwhile, some previous studies have shown that the level of oligomeric α-syn or phosphorylated a-syn (p-Asyn), two types associated with its toxicity mechanisms, is elevated in blood or CSF samples obtained from patients with PD (El-Agnaf et al., 2006; Ballard and Jones, 2010; Foulds et al., 2011). Various forms and shapes with equally diverse methods and low reproducibility between laboratories are the challenge issues, mainly as a result of the specific aggregation protocols used to synthesize these species. Due to the inconsistent results, further independent validation studies are needed. In contrast to PD and PD syndromes (PDs), due to overlapped symptoms and short of disease-specific biomarkers, the clinical differential diagnosis between PD and the atypical parkinsonian disorders turns out to be difficult, as reflected by associated high rates of misdiagnosis and limited precision, especially in early disease stages.

Application of p-Asyn (ser129), total α-syn, and oligomeric α-syn in the diagnosis, evaluation, and prognosis of PD have been reported in a large number of articles. In this study, we firstly aimed to explore the combined diagnostic value of plasma Lewy body-associated proteins (p-Asyn, total α-syn, and oligomeric α-syn) for the diagnosis of PD versus healthy controls (HCs) and other PDs; investigated correlations between these proteins and disease severity of patients with PD and clinical characteristics prediction.

## Materials and Methods

### Standard Protocol Approvals, Registrations, and Patient Consent

All patients in this cohort were consecutively enrolled in the clinical practice of the Department of Neurology of the First Affiliated Hospital of Wenzhou Medical University, a primary medical unit in Wenzhou, from October 2018 to September 2021, and underwent a standardized clinical assessment. The study was approved by the Institutional Ethics Board Committee of the Wenzhou Medical University First Affiliated Hospital on human experimentation before study initiation. All participants provided written informed consent prior to participating in this study. Our core research question was to determine whether plasma p-Asyn (ser129), total α-syn, and oligomeric α-syn levels could be candidate biomarkers for idiopathic PD diagnosis, as well as differentiate patients with PDs (e.g., MSA, PSP, or Vascular PD). The study was designed to provide evidence that plasma Lewy body-associated proteins levels could distinguish patients with PD from either HCs or those with PDs.

### Study Populations

**FIGURE 1:**
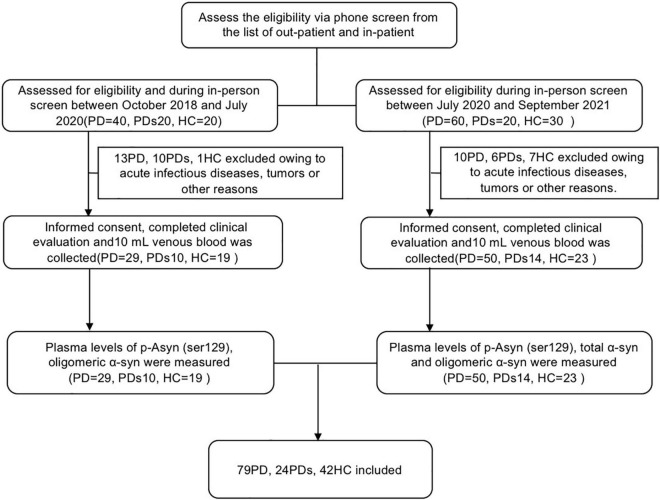
The flow chart shows the whole process of this study. PD was diagnosed according to the MDS clinical diagnostic criteria. Healthy controls were spouses and friends of patients with PD and were neurologically normal participants, including movement disorders or dementia. All healthy controls were recruited from the same institute. PDs comprised MSA, PSP, or Vascular PD. In all subjects, those who had other neurodegenerative diseases, such as Alzheimer’s disease (AD), Wilson’s disease (WD), acute infectious diseases, and tumors, were excluded. Clinical PD and PDs diagnoses were performed by experienced movement disorders specialty-trained neurologists. Plasma levels of p-Asyn (ser129) and oligomeric α-syn were measured in the first group (screen between October 2018 and July 2020). To further assess the diagnostic value of a-synuclein, the measurement of total α-syn was added in the second group (screen between July 2020 and September 2021).

Individuals included in the study were clinically featured using standard scales that evaluated neuro- logical, cognitive, and movement disorder components. As shown in Figure 1, this study included 145 participants: 79 patients with PD, 24 patients with PDs, and 42 HCs. PD was diagnosed according to the MDS clinical diagnostic criteria (Postuma et al., 2015). HCs were spouses and friends of patients with PD and were neurologically normal participants, including movement disorders or dementia. All HCs were recruited from the same institute. PDs comprised MSA, PSP, or VPD. In all subjects, those who had other neurodegenerative diseases, such as Alzheimer’s disease (AD), Wilson’s disease (WD), acute infectious diseases, tumors, etc., were excluded. Clinical PD and PDs diagnoses were performed by experienced movement disorders specialty-trained neurologists. All included subjects received assessments of motor and cognitive performance. Motor severity was evaluated by the Unified Parkinson’s Disease Rating Scale (UPDRS) part III motor scores. Cognition was examined by the Mini-Mental State Examination (MMSE).

### Clinical Evaluation

All subjects provided demographic information, medical history, and underwent physical and neurological examinations. Regarding the clinical assessment, the UPDRS modified Hoehn and Yahr stage, Hamilton Anxiety Scale (HAMA), Hamilton Depression Scale (HAMD), Activities of daily living (ADL), Rapid eye movement sleep behavior disorder questionnaire (RBDQ), and MMSE were used.

### Measurement of Plasma Biomarkers

Plasma levels of p-Asyn (ser129), total α-syn, and oligomeric α-syn were measured by enzyme-linked immunosorbent assay (ELISA). At enrollment, we collected 10 ml of venous blood from each subject in EDTA-plasma tubes. Blood samples were centrifuged (2,500 *g* for 15 min) within 1 h of collection. Following centrifugation, plasma 0.25 ml aliquots were stored in cryotubes at −80°C before testing. Plasma levels of p-Asyn (ser129), total α-syn, and oligomeric α-syn were measured with individual assay kits (Jianglai Biological Company, Shanghai, China; No: JL12589, JL12231, and JL41188, respectively) by research assistants who were blinded to the clinical diagnosis. In total, 80 μl of standard solution and 20 μl samples(5 × diluted) were pipetted into the wells of 96-well plates. In total, 100 μl of antibody-HRP conjugate (anti-p-Asyn (ser129) antibody, anti-total α-syn antibody, and oligomeric α-syn antibody; MyBioSource, USA) was added to standard wells and sample wells, covered with an adhesive strip and incubated for 60 min at 37°C. After 4 times washing, plates were incubated in TMB substrate for 15 min at 37°C, and reactions were stopped with 2 mol/l H_2_SO_4_. Plates were read at 450 nm. All samples were run in duplicate.

### Statistical Analyses

A continuous variable was shown as mean ± standard deviation, and a categorical variable was described as count and percentage. The continuous variable was compared using the analysis of variance or the non-parametric Kruskal–Wallis test or the Mann–Whitney *U* test, and the categorical variable was compared using the X^2^ test or Fisher’s exact test. The primary method for examining the discriminative performance of the biomarkers was the sensitivity, specificity, and area under the receiver operating characteristics curve (AUC). Bonferroni correction was applied to account for multiple comparisons. All studied subjects were included in correlation analyses to compare Lewy body-associated proteins and motor UPDRS Part III and Cognition MMSE scores. All the correlations coefficients were calculated by a two-tailed Spearman rank test, and all the individuals were considered for the study. GraphPad Prism software (version 8) was used to analyze the data. The significance level was set at *p* < 0.05.

## Results

### Baseline Phenotypic Characteristics of This Cohort

The study included 145 clinically diagnosed participants (Figure 1); of those, 79 patients (54.5%) had PD, 24 patients (16.6%) had PDs (MSA:9, VPD: 8, PSP:7), and 42 (29.0%) were HCs. Table 1 summarizes the demographic and clinical information for all participants. The mean age was 65.43 (SD, 7.467) in the control group, 64.49 (SD, 8.22) in participants with PD, and 69.25 (SD, 7.95) in PDs respectively. The 3 groups were not significantly different in education. Participants with PD had a median of 4.12 years (SD, 3.759) postdiagnosis, while 3.79 years (SD, 3.856) in participants with PDs. Patients with PD had a longer disease duration than patients with PDs (*p* < 0.0001). Table 1 showed the mean UPDRS scores, Hoehn–Yahr (H–Y) stages, MMSE, HAMA, HAMD, ADL, BMI, and RBDQ, as well as the levels of p-Asyn (ser219), total α-syn, and oligomeric α-syn, etc. Both patients with PD and PDs exhibited higher UPDRS, HAMD, HAMA, and ADL (*p* < 0.0001). For MMSE, the score was lower in both PD and PDs groups than in the controls (*p* = 0.002). There were no differences in terms of the majority neurological examinations scales between the PD and PDS groups, even though the sample size was small in the PDs group.

**TABLE 1:** Basic demographics and clinical characteristics of the cohort participants.

Characteristics	Control	PD	PDS	*p-value^a^*
Number	42	79	24	NA
Sex (Male: Female)	16:26	42:37	17:7	0.036*^b^*
Age [years], Mean (SD)	65.43 (7.467)	64.49 (8.224)	69.25 (7.952)	0.057
Duration [years], Mean (SD)	–	4.58 (3.849)	3.17 (3.315)	<0.0001
Education [years], Mean (SD)	3.76 (4.178)	4.12 (3.759)	3.79 (3.856)	0.682
H&Y stages, Mean (SD)	–	2.14 (0.974)	2.85 (1.048)	<0.0001
UPDRS, Mean (SD)	2.67 (3.986)	40.77 (17.746)	55.42 (27.204)	<0.0001
UPDRS-I, Mean (SD)	0.31 (0.517)	2.51 (2.124)	4.33 (4.429)	<0.0001
UPDRS-II, Mean (SD)	0.29 (0.742)	11.67 (6.091)	15.50 (7.846)	<0.0001
UPDRS-III, Mean (SD)	1.64 (3.655)	23.59 (11.709)	32.79 (16.922)	<0.0001
UPDRS-IV, Mean (SD)	0.44 (0.770)	2.85 (2.865)	2.79 (2.284)	<0.0001
MMSE, Mean (SD)	23.33 (4.65)	22.85 (5.897)	17.63 (7.216)	0.002
HAMD, Mean (SD)	3.31 (3.751)	7.09 (5.221)	7.83 (4.280)	<0.0001
HAMA, Mean (SD)	5.36 (4.95)	11.13 (7.436)	10.71 (6.003)	<0.0001
RBDQ-HK, Mean (SD)	6.83 (7.705)	24.13 (20.096)	23.46 (21.821)	<0.0001
ADL, Mean (SD)	20.29 (0.970)	26.70 (7.771)	38.88 (15.037)	<0.0001
BMI [kg/m^2^], Mean (SD)	24.26 (2.66)	24.88 (5.087)	24.60 (4.180)	0.925
p-Asyn ser129 (ng/ml), Mean (SD)	15.34 (2.042)	17.13 (3.055)	16.91 (2.264)	0.006
Total α-syn (ng/ml), Mean (SD)	27.55 (5.762)	34.95 (8.002)	33.70 (7.012)	<0.0001
Oligomeric α-syn (ng/ml), Mean (SD)	2.41 (0.512)	2.80 (0.540)	2.84 (0.411)	<0.0001

*^a^P-values obtained from Kruskal–Wallis test; ^b^P-values obtained from the chi-squared test.*

*PD, Parkinson’s Disease; PDS, Parkinsonian syndrome; SD, Standadized deviation; UPDRS, unified Parkinson’s disease rating scale; MMSE, mini-mental state examination; HAMD, Hamilton Depression Scale; HAMA, Hamilton Anxiety Scale; RBDQ-HK, REM sleep behavior disorder questionnaire – Hong Kong); ADL, activity of daily living scale; BMI, body mass index.*

## Lewy Body-Associated Proteins Were Elevated in the Plasma of Parkinson’s Disease Patients

**FIGURE 2:**
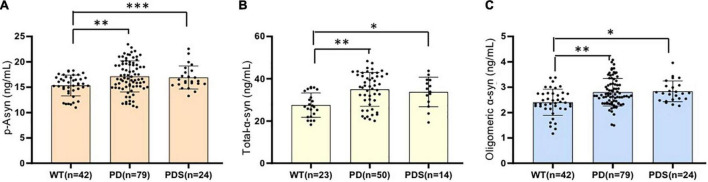
Lewy body-associated proteins are elevated in the plasma of patients with PD. Plasma p-Asyn at ser129 **(A)**, total α-syn **(B)**, and oligomeric α-syn **(C)** levels for healthy controls, patients with PD, and PDs included in this cross-sectional analysis. Mean plasma Lewy body-associated proteins levels were significantly increased in patients with PD and PDs compared to age-matched healthy controls (*p* < 0.05, Mann–Whitney *U* test). *P < 0.05, **P < 0.01, and ***P < 0.001.

**FIGURE 3:**
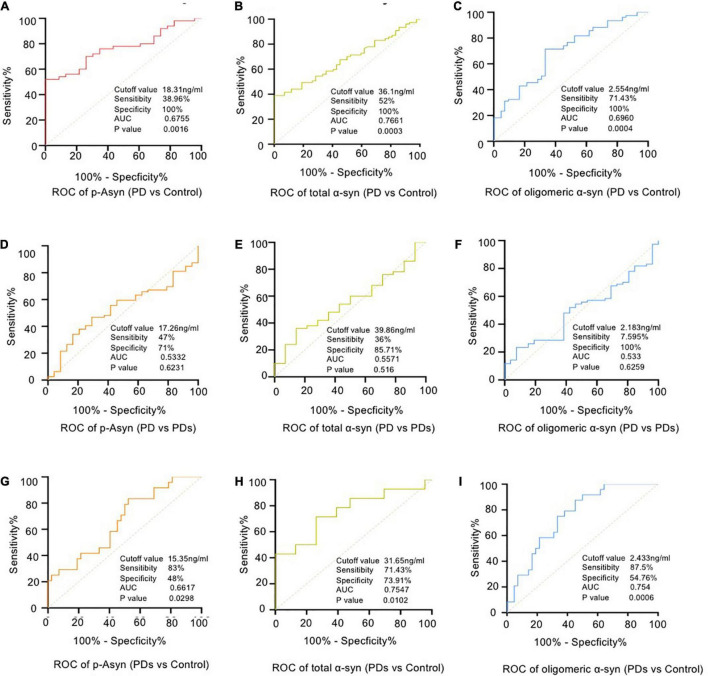
Receiver operating characteristic (ROC) curve analyses for differentiating between PD vs HCs **(A–C)**, PD vs PDs **(D–F)** and PDs vs HCs **(G–I)**. PD = Parkinson’s Disease; PDs = Parkinsonian syndrome; HCs = Healthy controls.

**FIGURE 4:**
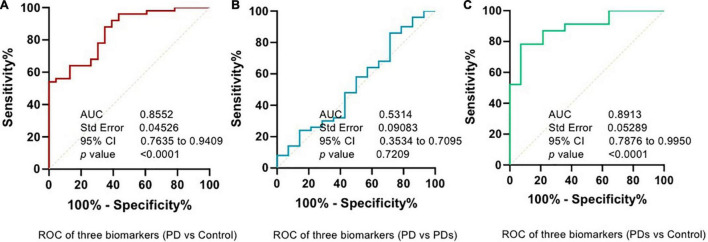
Combined assessment of a panel of plasma p-Asyn at ser129, total α-syn, and oligomeric α-syn is a better biomarker for Parkinson’s disease. **(A)** ROC curve of combined biomarkers between PD and HCs; **(B)** ROC curve of combined biomarkers between PD and PDs; **(C)** ROC curve of combined biomarkers between PDs and HCs.

The plasma p-Asyn levels were significantly higher in patients with PD (17.13 ± 3.055 ng/ml) than in age-matched HCs (15.34 ± 2.042 ng/ml, *p* = 0.0019, Table 1 and Figure 2A). Analogously, both total α-syn and oligomeric α-syn levels were apparently higher in PD (34.95 ± 8.002 ng/ml and 2.80 ± 0.540 ng/ml, respectively) than controls (27.55 ± 5.762 ng/ml, *p* = 0.0002; and 2.406 ± 0.513 ng/ml, *p* = 0.0005, respectively, Table 1 and Figures 2B,C). The aforementioned results showed that the plasma Lewy body-associated proteins levels were significantly higher in patients with PD than in HCs. However, there was no difference in patients with PD and PDs (Figures 2A–C). To identify whether Lewy body-associated proteins could be a plasma-based biomarker for PD, ROC analysis was performed and the primary outcome showed that plasma Lewy body-associated proteins had high sensitivity and specificity for distinguishing PD from controls, with an AUC of 0.6755 for p-Asyn (95%CI, 0.57–0.76, cutoff value: 18.31 ng/ml), 0.7661 for total α-syn (95%CI, 0.65–0.87, cutoff value: 36.1 ng/ml) and 0.6960 for oligomeric α-syn (95%CI, 0.64–0.82, cutoff value: 2.554 ng/ml), respectively (Figures 3A–C). As for plasma biomarkers that compared participants with PD and PDs, the AUC was 0.5332 for p-Asyn (95%CI, 0.57–0.76), 0.5571 for total α-syn (95%CI, 0.65–0.87), and 0.5065 for oligomeric α-syn (95%CI, 0.64–0.82), respectively (Figures 3D–F), indicating that it was unable to discriminate PD and PDs. Meanwhile, compared PDs with controls, the AUC was 0.6617 for p-Asyn(95%CI, 0.53–0.80), 0.7547 for total α-syn (95%CI, 0.58–0.93), and 0.7540 for oligomeric α-syn (95%CI, 0.64–0.87), respectively (Figures 3G–I). Of note, the combination of plasma p-Asyn, total α-syn, and oligomeric α-syn was a better biomarker for discriminating PD from HCs, with an AUC of 0.8552 (*p* < 0.0001, 95%CI, 0.7635–0.9409, Figure 4A), which was significantly higher than plasma p-Asyn (ΔAUC, 0.1797), total α-syn (ΔAUC, 0.0891) and oligomeric α-syn (ΔAUC, 0.1592) alone, suggesting that three markers offer partly unique and complementary insights to potential disease pathologies. Figure 4B showed the combination of three plasma biomarkers was unable to differentiate PD from PDs, with an AUC of 0.5314 (*p* = 0.7209). Between PDs and controls, with an AUC of 0.8913 (*p* < 0.0001, 95%CI, 0.7635–0.9409, Figure 4C) for the three biomarkers.

### Lewy Body-Associated Proteins Were Not Associated With Disease Duration and Severity in Patients With Parkinson’s Disease

**FIGURE 5:**
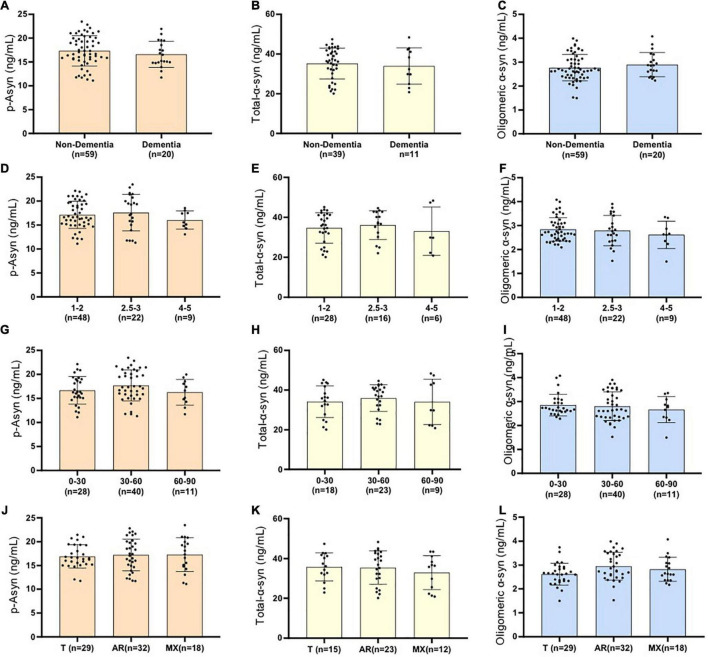
Examined whether plasma Lewy body-associated proteins levels were correlated with disease severity in terms of either motor Hoehn–Yahr stages **(A–C)**, cognitive symptoms **(D–F)**, UPDRS scores **(G–I)**, and subtypes of clinical symptoms **(J–L)** among patients with PD (*p* > 0.05, the Mann–Whitney *U* test and the Kruskal–Wallis test). No significant difference is presented as ns. Non-dementia in **(A–C)** represents PD patients without dementia; Dementia in **(A–C)** represents PD patients with dementia; Cognitive impairment was evaluated by MMSE (patients with MMSE no more than the cut point is defined as dementia; illiteracy at 17, primary school education at 20, high school degree and higher degree at 24); Patients with PD are divided into three groups with different motor Hoehn–Yahr stages(H–Y = 1–2, H–Y = 2.5–3, H–Y = 4–5) and different UPDRS scores(UPDRS = 0–30, UPDRS = 30–60, UPDRS>60). Using portions of the Unified Parkinson’s Disease Rating Scale, a ratio value was derived, yielding three patient subtypes: a tremor-dominant group (T), an akinetic-rigid group (AR), and a mixed group (MX).

As for idiopathic PD,whether age, disease duration, and severity might influence plasma Lewy body-associated proteins concentration need to be determined. We examined participants of various ages and disease duration and found plasma Lewy body-associated proteins concentrations were not correlated with age and disease duration in PD (Table 2). We next examined whether plasma Lewy body-associated proteins levels were correlated with disease severity in terms of either motor (H–Y stages) or cognitive symptoms (MMSE) among patients with PD. In assessing PD motor symptom severity, we observed that p-Asyn (ser129), total α-syn, and oligomeric α-syn levels had no significant difference through the H–Y stages increased (17.13 ± 2.832, 17.59 ± 2.823, 16.04 ± 1.899 ng/ml of p-Asyn, 34.68 ± 7.665, 36.12 ± 7.206, 33.08 ± 12.06 ng/ml of total α-syn, 2.839 ± 0.492, 2.792 ± 0.633, 2.613 ± 0.568 ng/ml of oligomeric α-syn, for H–Y stage through 1–5, Figures 5A–C). Regarding PD dementia severity, we tested the relationship between plasma Lewy body-associated proteins levels and various levels of cognitive ability. We found that p-Asyn, total α-syn, and oligomeric α-syn levels were similar as the severity of cognitive dysfunction increased (17.32 ± 3.162, 16.59 ± 2.718 ng/ml of p-Asyn, 35.23 ± 7.753, 33.95 ± 9.161 ng/ml of total α-syn, and 2.77 ± 0.551, 2.895 ± 0.507 ng/ml of oligomeric α-syn for cognition status between Non-dementia and dementia groups, Figures 5D–F). Notably, Lewy body-associated proteins had no relationship between different motor stages and dementia performances. Similarly, in terms of the UPDRS scores (Figures 5G–I) and subtypes of clinical symptoms (Figures 5J–L) among patients with PD, there was no significant difference.

**TABLE 2:** Correlation Lewy body-associated proteins with age and disease duration.

*Age (Years)*	<50 (*n* = 5)	50–60 (*n* = 17)	60–70 (*n* = 38)	>70 (*n* = 19)	*p-value^a^*
p-Asyn ser129	15.88 (2.373)	17.76 (2.645)	16.74 (3.263)	17.70 (3.104)	0.380
Oligomeric α-syn	2.806 (0.311)	2.904 (0.5685)	2.876 (0.507)	2.553 (0.5831)	0.270
***Age (Years)***	**<*50 (n*** = 4)	***50–60 (n* = *12)***	***60–70 (n* = *21)***	**>*70 (n* = *13)***	***p-value*** *^a^*
Total (α-syn	34.31 (10.54)	35.67 (7.396)	32.69 (8.536)	38.13 (6.443)	0.2397
***Disease duration (Years)***	**≤1 (*n*** = 15)	***1y–5y (n* = *42)***	***5y–10y (n*** ( = 12)	***>10y (n* = *8)***	***p-value*** *^a^*
p-Asyn ser129	17.80 (2.906)	16.90 (2.946)	16.05 (3.434)	18.77 (2.988)	0.264
Oligomeric α-syn	2.984 (0.508)	2.717 (0.557)	2.972 (0.474)	2.651 (0.533)	0.114
***Disease duration (Years)***	**≤*1 (n*** = 12)	***1y–5y (n* = *25)***	***5y–10y (n* = *9)***	***>10y (n*** = 4)	***p-value*** *^a^*
Total α-syn	36.24 (5.892)	35.15 (8.216)	29.32 (8.117)	42.46 (5.064)	0.041

*^a^P-values obtained from the Kruskal–Wallis test.*

### Correlation Between Lewy Body-Associated Proteins Levels and Clinical Characteristics Scores

We further examined whether plasma Lewy body-associated proteins levels were correlated with other clinical characteristics prediction in terms of UPDRS, HAMD, HAMA, ADL, RBD, etc. According to the Spearman correlation analysis results, plasma Lewy body-associated proteins concentrations did not correlate with the aforementioned clinical characteristics scores in PD (Table 3). Furthermore, when the multivariate regression analysis simultaneously considered age and sex, the correlation between Lewy body-associated proteins levels and clinical characteristics remained non-significant. These findings suggested that plasma Lewy body-associated proteins levels were not related to worse clinical performances in patients with PD.

**TABLE 3:** Partial correlation analysis between Lewy body-associated proteins levels and clinical characteristics scores.

Clinical scales	p-Asyn ser129 (ng/ml)	Total α -syn (ng/ml)	Oligomeric α -syn (ng/ml)
Total UPDRS	−0.155 (0.288)	−0.171 (0.240)	−0.084 (0.566)
UPDRS I	0.126 (0.390)	0.113 (0.439)	0.064 (0.663)
UPDRS II	−0.083 (0.573)	−0.161 (0.270)	−0.159 (0.275)
UPDRS III	−0.210 (0.148)	−0.176 (0.225)	−0.035 (0.811)
UPDRS IV	−0.026 (0.859)	−0.096 (0.513)	−0.100 (0.494)
Hoehn–Yahr stage	−0.272 (0.059)	−0.223 (0.108)	−0.093 (0.526)
MMSE	0.204 (0.160)	0.225 (0.119)	−0.192 (0.185)
HAMD	0.105 (0.474)	0.079 (0.587)	0.025 (0.863)
HAMA	−0.048 (0.745)	0.076 (0.604)	0.084 (0.564)
RBDQ-HK	0.036 (0.806)	−0.122 (0.402)	−0.141 (0.334)

*UPDRS, unified Parkinson’s disease rating scale; MMSE, mini-mental state examination; HAMD, Hamilton Depression Scale; HAMA, Hamilton Anxiety Scale; RBDQ-HK, REM sleep behavior disorder questionnaire – Hong Kong.*

## Discussion

In the present study, we demonstrated that plasma p-Asyn (ser129), total α-syn, and oligomeric α-syn levels in plasma were significantly higher in patients with clinically diagnosed PD compared with HCs, which may serve as diagnostic biomarkers in PD. Meanwhile, we found that combined plasma p-Asyn, total α-syn, and oligomeric α-syn was a better biomarker for discriminating PD from controls, with an AUC of 0.8552. However, there was no significant difference between the PD and PDs groups. Of note, increased Lewy body-associated proteins were not associated with a higher risk of motor symptoms (UPDRS III, H–Y stages), cognition features (MMSE), and other clinical characteristics scores, including HAMA, HAMD, RBD, etc.

Due to the invasive feature of lumbar puncture for CSF collection, it was not practical to gather CSF for routine operations during clinical visits. Moreover, lumbar puncture for CSF sampling was not always available because of the associated contraindications and participants’ consent. Hence, there was a need for non-invasive fluid biomarkers in PD, such as plasma, which was easier to perform and much less invasive, particularly in early disease stages with the highest rate of misdiagnosis. To our knowledge, α-syn played a key role in the occurrence and development of PD and was one of the core factors in the pathogenesis of PD, particularly p-Asyn (ser129) and oligomeric α-syn. Afterward, this paper chose to focus on exploring the value of α-syn as biomarkers. For the first time, this study gave the idea to test the plasma p-Asyn, total α-syn, and oligomeric α-syn levels together and analyze the AUC for diagnosis accuracy in individuals with PD and HCs. Generally, patients with PD only get routine blood tests at present. Special blood tests for PD-specific indicators such as total α- syn, p-Asyn (at a different locus), oligomeric/poly α-syn, etc., were supposed to be carried out. Recently, in the biomarkers field, plasma-based analyses (e.g., tau 217) provided comparable diagnostic sensitivity and specificity to CSF-based assays (Palmqvist et al., 2020), supporting the confidence to promote the study. Notably, at present, there is no effective diagnostic test to reliably differentiate PD subjects from living patients with PDs. Consistent with this condition, we observed similar negative results between PD and PDs when using Lewy body-associated proteins as a discriminative indicator. PDs are defined as diseases with partial or whole symptoms of Parkinson’s disease, including MSA, PSP, VPD, etc., of which many types also had abnormal changes of SNCA, accounting for the difficulty to completely distinguish PD from PDs. On the other hand, since the cases of PDs were much fewer than participants with PD, more studies and bigger samples are necessary to validate this finding.

In individuals with PD, pathological α-syn aggregation in the brain began years before the symptoms of the disease became evident. Increased α-syn concentration in the plasma was supposed as a biomarker of PD pathology (Atik et al., 2016). A-syn was a cytoplasmic protein that appeared as an unstructured monomer or moderate stable tetramer (Surguchov, 2015). Under pathological stress, its conformation changed from a random coil to form unstable oligomers and protofibrils, then followed by fibrils until insoluble protein aggregates formed (Singleton et al., 2003), which were the toxic forms of the a-syn. In addition, phosphorylation at S129 (p-S129) was the most discussed post-translational modification in a-syn, its role was still unclear in terms of modulating a-syn (Sato et al., 2013). Post-translational modifications of the protein, such as phosphorylation or ubiquitination, could play a role in facilitating protein misfolding (Tian et al., 2019). Numerous investigations in biomarkers, therefore, focused on the measurement of total α-syn first in CSF and blood, and specific α-syn species (e.g., oligomeric α-syn, p-Asyn at residue Ser129) had also been considered as potential diagnostic biomarkers for PD. In this study, we found that plasma p-Asyn, total α-syn, and oligomeric α-syn levels were significantly higher in patients with PD, and three biomarkers together could distinguish PD from controls with an AUC of 0.8552, which was comparable with plasma neuronal exosome a-syn for PD (Niu et al., 2020). In line with this finding, studies on plasma oligomeric α-syn provided unanimous results, showing increased quantities in patients with PD (Williams et al., 2016). Moreover, plasma p-Asyn was higher in patients with PD compared with controls with an AUC of 0.71 (Schmid et al., 2013), similar to our result (AUC 0.6755). Of note, a panel of post-translational modified forms of α-syn, including p-Asy,n Tyr125, nitrated α-syn, glycated α-syn, and SUMOylated α-syn, showed high discriminatory power in distinguishing patients with PD (AUC 0⋅84; Vicente Miranda et al., 2017). In this study, we found a panel of plasma p-Asyn (ser129), total α-syn, and oligomeric α-syn can differentiate PD from controls. However, several previous studies reported that plasma a-syn may decrease, increase, or have no difference in patients with PD compared with controls (Besong-Agbo et al., 2013; Andersen et al., 2017; Lin et al., 2017; Vermeiren et al., 2020). These results, together with other factors including plasma storage condition, disease staging, sample preparation, etc., limited the utility of plasma total α-syn as a biomarker. Larger sample size may be needed to obtain more convincing results.

In addition, our findings extended current knowledge by showing that a panel of plasma p-Asyn, total α-syn, and oligomeric α-syn levels were not correlated with UPDRS III, MMSE, and other clinical characteristics scores of patients with PD, suggesting that this measurement could not be used as an indicator for reflecting disease severity in both motor and non-motor symptoms of PD. Several previous studies reported that α-syn was not associated with the severity of PD clinical symptoms. One of the probabilities may be that each individual had different sensitivity and tolerance to α- Syn. In addition, the plasma α- syn value was not directly equal to its deposition of each target organ. Therefore, SNCA was only able to diagnose PD rather than grade the severity. When it came to this study, usually participants had regular drug control during the evaluation period, causing difficulty to avoid the influence of drugs.

## Limitations

This study had several limitations. For instance, compared with PD and HCs, the number of cases with PDs, including MSA, PSP, and VPD, was relatively less. Our findings have to be further verified with a large number of participants. Another limitation of our study was that the sensitivity and specificity of plasma test assays for Lewy body-associated proteins from living patients were mainly based on the clinical diagnoses of PD and PDs and could be highly variable. Furthermore, the major disadvantages of this study were that we only used cross-sectional study designs and lacked prospective follow-up with repeated plasma collection and clinical assessments. Finally, immunocapture techniques, which representatively adopted paired antibodies in the sandwich immunoassay format, were still the most common skills for the majority of biomarker analyses. However, these methods used in this study, including ELISA, were influenced by the limitations of the antibody-based capture methods, for example, variability in dynamic range, restricted antibody specificity, the existence of cross-reactivity, etc.

## Conclusion

In summary, our results suggested that a panel of plasma Lewy body-associated proteins, including p-Asyn (ser129), total α-syn, and oligomeric α-syn, may serve as a non-invasive biomarker to aid the diagnosis of PD from HCs. In addition, increased plasma Lewy body-associated proteins were not associated with the progression of motor and non-motor symptoms, opposing its potential role as a prognostic marker for PD progression.

## Data Availability Statement

The raw data supporting the conclusions of this article will be made available by the authors, without undue reservation.

## Ethics Statement

The studies involving human participants were reviewed and approved by the study was approved by the institutional Ethics Board Committee of the Wenzhou Medical University First Affiliated Hospital on human experimentation before study initiation. The patients/participants provided their written informed consent to participate in this study.

## Author Contributions

HH and XZ collected the patients, analyzed the data and, helped to revise the manuscript. XX, FF, QY, and SZ made substantial contributions to the conception of the manuscript and replenished the required data. CX and KX were involved in drafting the manuscript. All authors contributed to the article and approved the submitted version.

## Conflict of Interest

The authors declare that the research was conducted in the absence of any commercial or financial relationships that could be construed as a potential conflict of interest.

## Publisher’s Note

All claims expressed in this article are solely those of the authors and do not necessarily represent those of their affiliated organizations, or those of the publisher, the editors and the reviewers. Any product that may be evaluated in this article, or claim that may be made by its manufacturer, is not guaranteed or endorsed by the publisher.
